# The role of muscle mass in vascular remodeling: insights from a single-leg amputee model

**DOI:** 10.1007/s00421-022-05076-1

**Published:** 2022-11-11

**Authors:** Anna Pedrinolla, Valentina Cavedon, Chiara Milanese, Chiara Barbi, Gaia Giuriato, Fabio Giuseppe Laginestra, Camilla Martignon, Federico Schena, Massimo Venturelli

**Affiliations:** 1 Section of Movement Science, Department of Neuroscience, Biomedicine, and Movement Science, University of Verona, Via Felice Casorati 43, 37137 Verona, Italy; 2 Division of Geriatrics, Department of Internal Medicine, University of Utah School of Medicine, Salt Lake City, Utah USA

**Keywords:** Vascular remodeling, Arterial diameter, Muscle mass, Amputee

## Abstract

**Purpose:**

Both muscle mass and physical activity are independent mechanisms that play a role in vascular remodeling, however, the direct impact of muscle mass on the structure and function of the vessels is not clear. The aim of the study was to determine the impact of muscle mass alteration on lower limbs arterial diameter, blood flow, shear rate and arterial stiffness.

**Methods:**

Nine (33 ± 13 yrs) male individuals with a single-leg amputation were recruited. Vascular size (femoral artery diameter), hemodynamics (femoral artery blood flow and shear rate were measured at the level of the common femoral artery in both amputated (AL) and whole limbs (WL). Muscle mass of both limbs, including thigh for AL and thigh and leg for WL, was measured with a DXA system.

**Results:**

AL muscle mass was reduced compared to the WL (3.2 ± 1.2 kg vs. 9.4 ± 2.1 kg; *p* = 0.001). Diameter of the femoral artery was reduced in the AL (0.5 ± 0.1 cm) in comparison to the WL (0.9 ± 0.2 cm, *p* = 0.001). However, femoral artery blood flow normalized for the muscle mass (AL = 81.5 ± 78.7ml min^−1^ kg^−1^,WL = 32.4 ± 18.3; *p* = 0.11), and blood shear rate (AL = 709.9 ± 371.4 s^−1^, WL = 526,9 ± 295,6; *p* = 0.374) were non different between limbs. A correlation was found only between muscle mass and femoral artery diameter (*p* = 0.003, *R* = 0.6561).

**Conclusion:**

The results of this study revealed that the massive muscle mass reduction caused by a leg amputation, but independent from the level of physical activity, is coupled by a dramatic arterial diameter decrease. Interestingly, hemodynamics and arterial stiffness do not seem to be impacted by these structural changes.

## Introduction

Vascular remodeling refers to the changes in structure and function of vessels, mostly due to the ability of the vessels to transduce the dynamic interaction between locally generated growth factors, vasoactive substances and hemodynamic changing within the physiological environment (Gibbons & Dzau 1994; Renna et al. 2013; Thijssen, Green and Hopman 2011). In the case of arteries, this adaptability is essential to successfully handle vascular distress, variations in metabolic activity, or arterial disease allowing vessels to adapt positively to physiological requirements (Renna et al. 2013). The arterial wall is made of endothelium cells, smooth muscle cells, and fibroblasts that interact with each other forming an autocrine-paracrine complex (Renna et al. 2013). In this way, the vascular wall detects changes in the environment being capable of integrating these intercellular communication signals and through the local production of mediators influences vascular structure and function (Renna et al. 2013). One of the most important determinants of vascular remodeling is the hemodynamic stimuli, a single term that includes shear rate, blood flow, and blood pressure, that stimulates growth factors and vasoactive substances supporting arterial adaptations (Green, Hopman, Padilla, Laughlin and Thijssen 2017; Renna et al. 2013; Thijssen et al. 2011). Indeed, the lumen of the vessel increase in association with long-term increases in flow as well as it may decrease in response to long-term decrease in flow. Also, shear-stress (known as the tractive force on endothelial cells induced by blood flow) activates a flow-sensitive potassium channel that induces hyperpolarization and promotes calcium influx, altering vascular tone (Gibbons and Dzau 1994). Current literature provides evidence about vascular remodeling coming from studies focused on the effect of physical inactivity in healthy individuals, but this model does not reflect the typical human behavior since it induces a sudden and extreme physical inactivity and a consequent dramatic change in hemodynamics, especially in the shear rate (Green et al. 2017; Pedrinolla et al. 2020a, b; Thijssen et al. 2011), as well as reduction in muscle mass together with several other physical inactivity side effect. Another model used to investigate vascular remodeling is the study of individuals with spinal cord injury (SCI) (Thijssen, Green and Hopman,^2011^; Venturelli et al. 2014). Investigators have found that below the spinal lesion, individuals with SCI present vascular dysfunction and smaller vessel size (Olive et al. 2003). Nonetheless, normalizing arterial size and hemodynamics for muscle mass, evidence of increased vascular responsiveness in this population was found, suggesting that muscle mass plays a key role in this process (Venturelli et al. 2021a, b). In light of that, doubts on the mechanisms involved in the process of vascular remodeling were raised: what if not only hemodynamics affects vascular remodeling? In support of this, it has to be considered that the vascular circuits are physiologically designed to meet the specific demands of their tissues, in terms of range and how blood flow is supplied to them (Folkow 1971). The consequent adaptation of its vasculature in terms of function and structure may be expected whether the tissue around blood circuits changes, and the microcirculation in the tissue sorraounding vessels changes. Furthermore, the SCI model has two limits: first, it includes a sudden lack of physical activity, and second, denervation rather than, or together with physical inactivity might play a critical role in this process (Thijssen et al. 2011). Therefore, finding new models to investigate this mechanism that do not include sudden and extreme physical inactivity or denervation may elucidate the role of muscle mass on vascular remodeling.

Thus, we recruited a group of single transtibial-amputee individuals and measured vascular size and hemodynamics at the common femoral artery in both amputated and whole limbs. The peculiarities of this model are many. First, the amputated leg is reduced in muscle mass compared with the contralateral leg. Second, it is still innervated and used for locomotion, so the effect of physical inactivity is not a limiting factor. Therefore, the aim of this study was to investigate vascular remodeling on a new human model, excluding physical inactivity and denervation, considering the role of muscle mass. We hypothesized that the same individual vascular structure and hemodynamics in the amputated-limb would be decreased compared to the whole leg, resulting in a smaller artery diameter together with a limb-specific difference in shear rate, blood flow, vascular conductance and stiffness. Also, we hypothesized that artery diameter would be positively correlated with muscle mass, and that once blood flow was normalized for muscle mass, there would be no differences in hemodynamics between the amputee and the whole limb.

## Methods

### Participants

The recruitment for the study started contacting previously known amputated individuals who already took part in other data collections at our department. Male individuals were included in the study if subjected to a traumatic transtibial amputation and if they were free of any other related comorbidity. People were also included in the study in the absence of pharmacological therapy, smoking history, atherosclerotic vascular disease, heart failure, and liver, renal, or inflammatory and metabolic diseases. All experiments were conducted after informed and written consent was obtained from the subjects in accordance with the Declaration of Helsinki, as part of a protocol approved by the Institutional Review Board of the Department of Neurosciences, Biomedicine, and Movement Sciences, University of Verona, Italy (Verona, Italy—#CT 27,111; NIH Clinical trial identification number: NCT03963050).

### Study overview

All assessment procedures were performed in the morning between 8.00 and 12.00 am. Upon arrival to the Human Anatomy and Exercise Physiology laboratories, subjects were measured for body mass and stature, and underwent the body composition assessment using a dual-energy x-ray absorptiometry (DXA). Following the DXA, subjects were placed supine on a bed and asked to rest for at least 15 min, and blood pressure was measured. Subsequently, measures of arterial diameter, blood velocity and flow were taken. All measurements were taken in both right and left femoral arteries (whole and amputated legs). Since common femoral artery supplies the whole lower limb, for the between limbs comparison the volume of the entire leg was used (thigh + lower leg for the whole leg and thigh + what was remaining of the lower leg for the amputated leg).

### Body composition and limb muscle mass

The fat-free soft tissue mass (FFSTM) was assessed by means of DXA using a total body scanner (QDR Horizon, Hologic MA, USA; fan-beam technology, software for Windows XP version 13.6.). In our laboratory, quality control of the DXA scanner is performed daily before actual use through an encapsulated spine phantom (Hologic Inc, Bedford, MA) to check for possible baseline drift. The subjects undertook whole-body DXA scanning according to “The Best Practice Protocol for the assessment of whole-body body composition by DXA” (Nana, Slater, Stewart and Burke, 2015). DXA scanning took place in the late morning, after a 3–4 h fast. All subjects were required not to undertake strenuous physical activity the day before the measurement session, as well as any exercise on the morning of the measurements. Subjects were asked to wear minimal clothing (i.e., underwear) and to void their bladder before having their body weight and stature measured. Body weight was assessed with the prosthesis to the nearest 0.1 kg using a certified electronic scale (Tanita electronic scale BWB-800 MA, Wunder SA.BI. Srl, Milano, Italy). The weight of the prosthesis was then taken and subtracted from the previous weight with the prosthesis to get the actual body weight. Standing height was measured to the nearest 0.1 cm using a Harpenden portable stadiometer (Holtain Ltd., Crymych, Pembs. UK) according to conventional criteria and measuring procedures (Lohman, Roche and Martorell 1992). Prior to scanning, subjects removed their prostheses and all metal, jewelry or reflective material. Positioning aids to support the residual lower limb were employed, and special strapping was applied around the subjects’ residual ankle to ensure no movement during the scans.

Analysis of DXA scans was performed according to the manufacturer’s procedures to get the whole-body FFSTM (expressed in grams). Additionally, the DXA scans were examined to define the left and right thigh and leg sub-regions according to Hart and colleagues (Hart, Nimphius, Spiteri, Cochrane and Newton 2015). The thigh region was delineated by a proximal boundary formed by an oblique line passing through the femoral neck to a distal boundary formed by the horizontal line passing through the knee axis, noted as the space between the femoral and tibial condyles. The proximal boundary of the leg region was a horizontal line passing through the knee axis as described above, while the distal boundary was a horizontal line spanning beneath the medial and lateral malleoli.

The same trained operator carried out all measurements and analyzed all the DXA scans to ensure consistency.

### Arterial diameter, blood velocity, shear rate and blood flow

Ultrasound Doppler (GE Logiq-7, General Electric Medical Systems, Milwaukee, WI, USA) was used for a 30 s recording of the common femoral artery, 2 cm above the bifurcation, both WL and AL, with the subject supine and at rest for at least 15 min. Rest arterial diameter, blood velocity, shear rate, and blood flow were determined for each subject. Arterial diameter was measured as the distance (mm) between the intima-lumen interfaces for the anterior and posterior walls in the arteries. Shear rate was calculated using arterial diameter and blood velocity (V_mean_) according to this formula (Pedrinolla, Venturelli, et al. 2020a):

$$ {\text{Shear rate }}\left( {{\text{s}}^{{ - {1}}} } \right) \, = { 8} \cdot {\text{V}}_{{{\text{mean}}}} /{\text{vessel diameter}} $$

Blood flow was calculated using arterial diameter and blood velocity according to this formula (Pedrinolla, Venturelli, et al. 2020b):

$$ {\text{Blood Flow }}\left( {{\text{ml}}\,{\text{min}}^{{ - {1}}} } \right) \, = {\text{ V}}_{{{\text{mean}}}} \cdot {\text{II}} \cdot \left( {{\text{vessel diameter}}/{2}} \right)^{{2}} \cdot {6}0 $$

Blood flow was normalized for the lower limb muscle mass volume obtained from DXA.

### Mean arterial pressure (MAP) and conductance

Arterial blood pressure was measured by means of a manual sphygmomanometer (Intermed, Milano, Italy) with the subject at rest and supine for at least 10 min. Arterial blood pressure was measured before the vascular tests.

MAP was calculated using systolic and diastolic blood pressure with the following formula:

$$ {\text{MAP }}\left( {{\text{mmHg}}} \right) \, = {\text{ Diastolic blood pressure }} + \, (\left( {{\text{Systolic blood pressure }} - {\text{ Diastolic blood pressure}}} \right)/{3} $$

Vascular conductance (VC) was then calculated with the following formula (Massimo Venturelli et al. 2021a, b):

$$ {\text{VC }}({\text{ml}}\,{\text{min}}^{{ - {1}}} \,{\text{mmHg}}^{{ - {1}}} ) \, = {\text{ Blood flow}}/{\text{MAP}} $$

*Statistical analysis*. 

### Statistical analysis

Data are expressed as mean ± SD. Paired t-tests were used to identify between-group differences (WL vs. AL). A Pearson’s analysis was used to identify correlations between vascular variables (diameter, blood velocity, shear rate, blood flow, and conductance) and limbs muscle mass. α level was set at 0.05. Cohen’s D was used to assess effect size, considering 0.2 small effect size, 0.5 moderate effect size, and 0.8 large effect size. All statistical analyses were performed with SigmaPlot Windows Version 14.0 (Systat Software, Chicago, IL).

## Results

### Participant characteristics

Nine single-leg amputee male individuals (33 ± 13 years old, 65 ± 12 kg, 14 ± 9 years since amputation) were included in the study. All subjects presented a traumatic transtibial amputation (*n* = 3 right-side, *n* = 6 left-side) and were free of any medications. Table 1 shows the characteristics of the subjects. All subjects declared to be involved in exercise acitivities at least three times a week (i.e.: soccer, running, cycling).

**Table 1:** Subjects’ characteristics

Variable	*N* = 9
Gender—M/F	9/0
Age—years	33 ± 13
Weight—kg	65 ± 12
Height—cm	171 ± 5
Systolic blood pressure—mmHg	126 ± 13
Diastolic blood pressure—mmHg	70 ± 7
Mean arterial pressure—mmHg	88 ± 8
Amputation	
For—years	14 ± 9
Side—(R/L)	3/6
Transtibial—n	9
Anthropometry	
Whole body FM—%	20.2 ± 5.4
Whole body FFM—%	76.2 ± 5.1
WL FM—%	21.4 ± 5.2
AL FM—%	34.2 ± 14.7
WL FFM—%	74.9 ± 4.9
AL FFM—%	63.5 ± 14.3

Data are presented as mean ± standard deviation. No statistical difference was found in anthropometric variables between WL and AL

*R/L* right/left-hand amputee, *WL* whole limb, *AL* amputated limb, *FM* fat mass, *FFM* free-fat mass

### Difference between whole limb and amputee limb

**Fig. 1:**
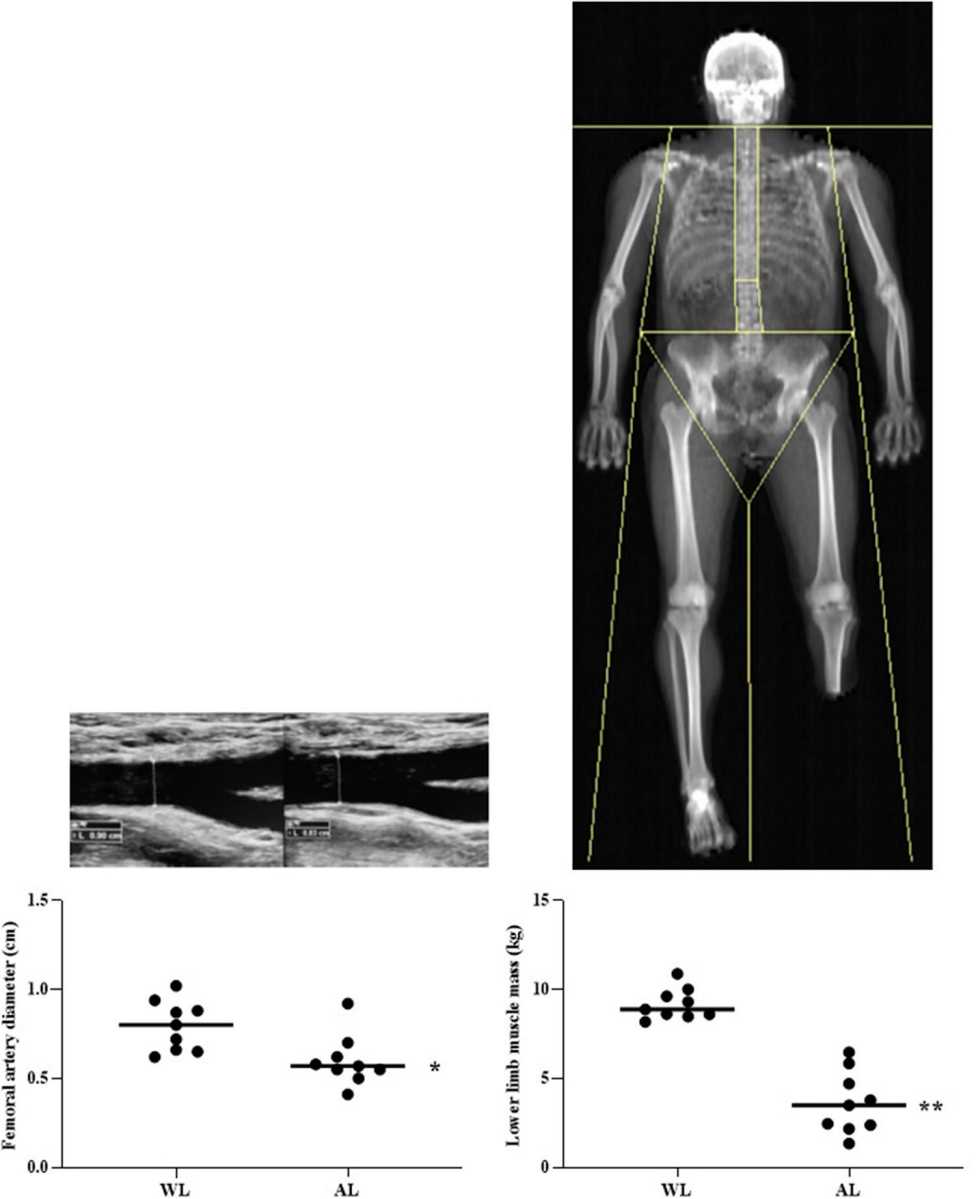
Structural variables. Femoral artery diameter (panel **A**) and muscle mass (panel **B**) in whole (WL) and Amputated(AL) limbs. *p < 0.005; **p < 0.001

**Fig. 2:**
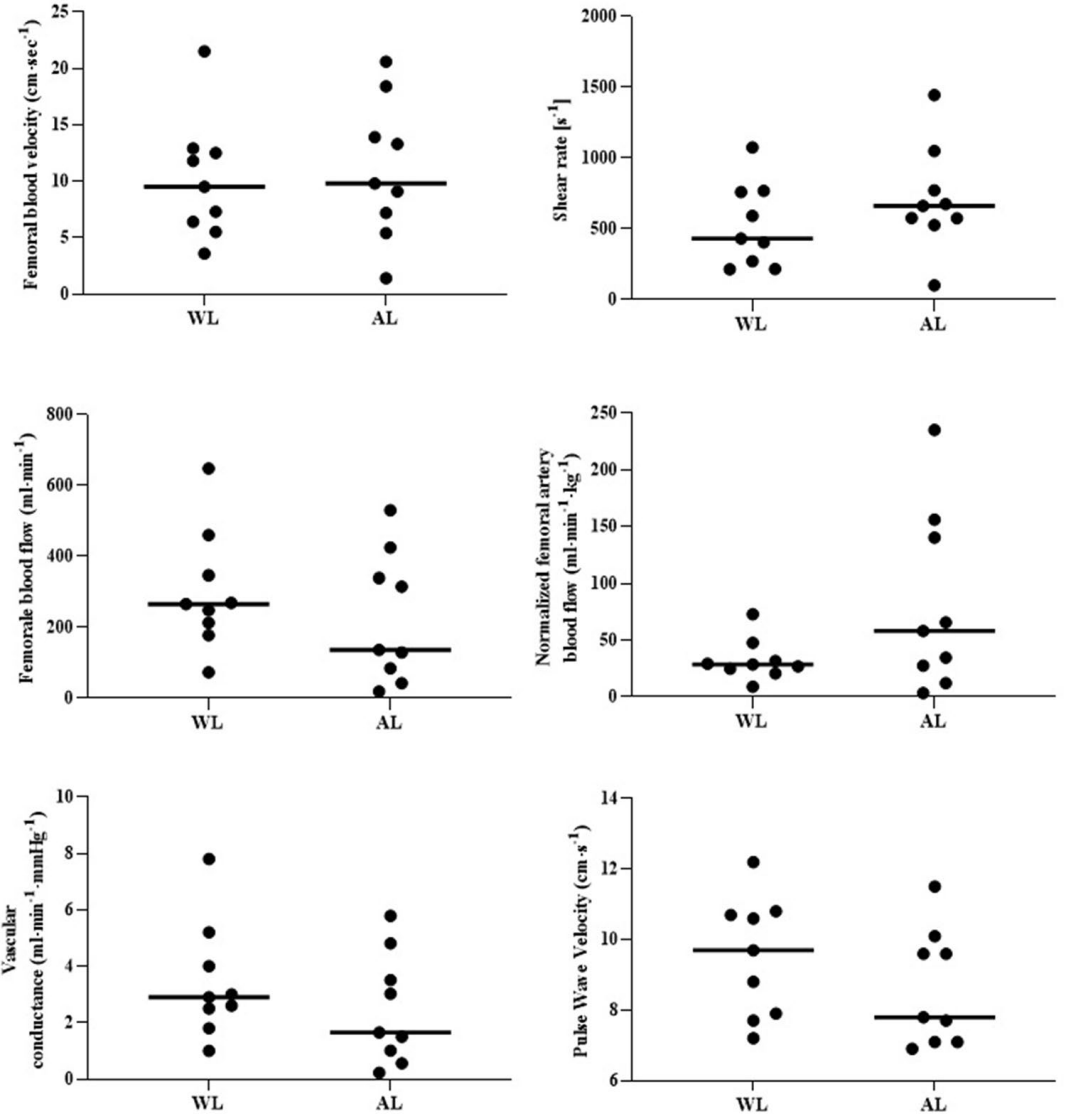
Vascular variables. Femoral blood velocity (panel **A**), shear rate (Panel **B**), femoral blood flow (panel **C**), normalized femoral blood (Panel **D**), vascular conductance (panel **E**), and carotid-femoral pulse wave velocity (panel **F**) in whole (WL) and amputated (AL) limbs

Arterial diameter was significantly smaller in the AL compared with WL (WL = 0.80 ± 0.1 cm, AL 0.62 ± 0.1 cm, -24%, *p* = 0.001) (Table 2; Fig. 1, Panel A). Also, muscle mass was significantly reduced in AL compared with WL (WL 9.1 ± 0.8 kg, AL 3.7 ± 1.8 kg, −59%, *p* < 0.001) (Table 2; Fig. 1, Panel B). No statistical differences were found between common femoral arteries of whole limb (WL) and amputee limb (AL) concerning of blood velocity (*p* = 0.766; Fig. 2, panel A), shear rate (*p* = 0.374; Fig. 2,panel B) blood flow (*p* = 0.385; Fig. 2, panel C), normalized blood flow (*p* = 0.115; Fig. 2, Panel D), and vascular conductance (*p* = 0.343; Fig. 2, Panel E) (Table 2).

**Table 2:** Paired t test between whole and amputee limbs for vascular variables and muscle mass

	AL	WL	Diff. of mean	*t*	*p*	Cohen's d
Femoral artery diameter—cm	0.8 ± 0.1	0.6 ± 0.1	−0.195	4.94	0.0011	0.7531
Femoral artery blood velocity—cm s^−1^	10.1 ± 5.4	11.1 ± 6.6	0.900	0.30	0.7663	0.0116
Shear rate—s^−1^	526.9 ± 295.6	709.9 ± 371.4	183.1	1.09	0.3743	0.1298
Femoral artery blood flow—ml min^−1^	329.7 ± 160.2	266 ± 184.3	−75.720	0.92	0.3858	0.0951
Femoral artery normalized blood flow—ml min^−1^ kg^−1^	32.5 ± 18	84.4 ± 83.6	49.07	1.76	0.1159	0.2798
Vascular conductance—ml min^−1^۰ mmHg^−1^	3.4 ± 2.1	2.6 ± 2.1	−0.96	1.00	0.3435	0.1125
Lower limb muscle mass—kg	9.1 ± 0.8	3.6 ± 1.7	−5.55	11.21	< 0.0001	0.9401

### Correlation between variables and limb muscle mass

**Fig. 3:**
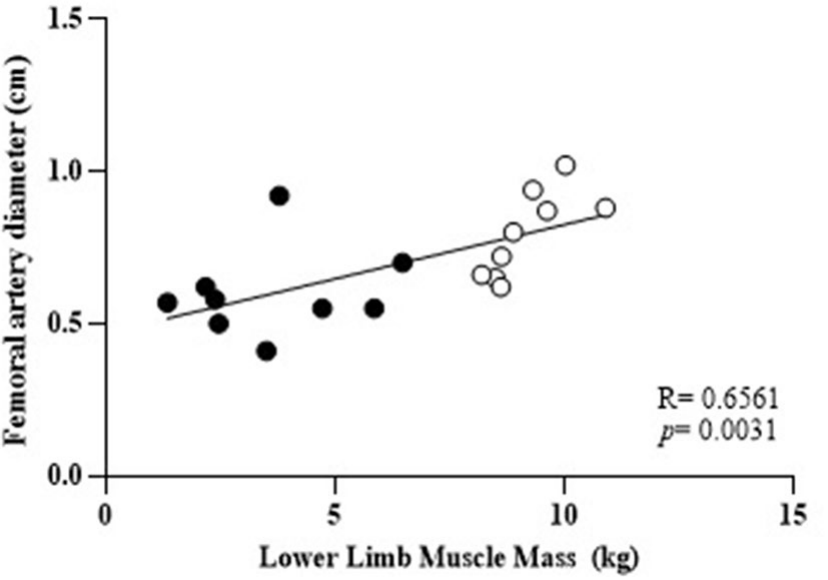
Correlation between structural variables: femoral artery diameter and lower limbs muscle mass. Whole limb (○) and amputated limb (●). *p* = 0.003, *r* = 0.6561

Pearson’s analysis shows a significant correlation only between muscle mass and femoral artery diameter (*r* = 0.6561; *p* = 0.003), Fig. 3 shows the correlations between these variables. No correlation was found between muscle mass and shear rate (*r* = 0.152, *p* = 0.109), muscle mass and blood flow (*r* = 0.058, *p* = 0.333), and muscle mass and vascular conductance (*r* = 0.076, *p* = 0.266).

## Discussion

Although mechanisms involved in vascular remodeling are the focus of many studies, to our knowledge this is the first study that centers the attention on the role of muscle mass in this process. Specifically, this is the first study considering a model that includes neither a sudden loss of physical activity (i.e., bed rest), nor denervation (i.e., individuals with SCI). Indeed, when using physical inactivity to study vascular remodeling, which may lead to reduction in muscle mass as well, actually there are several other physiological events happening and so what can be seen at vascular level would not be just the result of inactivity it self, but also the result of the side effect of bed rest. The same when looking at people with SCI, who experienced traumatic denervation, together with sudden extreme loss of physical inactivity and they live with side effects of this situation and most of the time they are taking pharmacological therapy. Using single-leg amputee model tostudy vascular remodeling, we wanted to investigate what happen ad vascular level when muscle mass is reduced, but in an individual who is still healthy and still regularly moving. We measured common femoral artery diameter, and hemodynamics in both limbs of single-leg amputee individuals regularly ambulating and performing physical activity with both lower limbs. The benefit of using single-leg amputee model is that this model allows to investigate hemodynamic stimuli in the same individual, in a model where the only difference between limbs is the muscle mass not as a consequences of some disease or particular conditions, and in the absence of neural involvement. The main finding of this study was that common femoral artery diameter significantly correlates exclusively with lower limbs muscle mass and not with other hemodynamic variables, confirming the initial hypothesis. Besides, our findings show that blood velocity, shear rate, blood flow, and vascular conductance do not differ between amputee and whole limbs. These results suggest that a reduction in muscle mass supplied by an artery, stimulates modification in the higher vessel tree.

### Evidence about the role of muscle mass in vascular remodeling

As previously mentioned, most of the evidence about structural arterial changes in humans includes models of physical inactivity or denervation associated with lack of movement (P. C. E. De Groot, Poelkens, Kooijman and Hopman 2004; Thijssen et al. 2011; Venturelli et al. 2021a, b). On the contrary, other researchers observed vascular remodeling in response to exercise, studying the mechanisms associated with the positive adaptation of vascular structure and function (Green et al. 2017; Thijssen et al. 2011). However, both ways to approach this physiological phenomenon (administering physical activity or inactivity), lead to the fact that the main factor involved in this process is an appropriate blood flow supply, which can guarantee proper shear stress, or else the frictional force of the blood cells on the endothelial layer (Groot et al. 2006). Indeed, shear stress is known to play a central role in endothelial function, nitric oxide bioavailability and vascular adaptation processes (Pedrinolla et al. 2020a, b; Pedrinolla et al. 2021), all variables that modulate vascular function (Tinken et al. 2010). Nonetheless, recent studies began to consider the muscle mass as a factor involved in the vascular remodeling process, but still, the model used in these studies included denervation and lack of physical activity that may bias the interpretation of the results (Venturelli et al. 2015; Venturelli et al. 2021a, b). Venturelli et al. (Venturelli et al. 2014) measured leg blood flow at the common femoral artery during passive leg movement in individuals with SCI and a control group showing an expected reduced absolute blood flow in SCI compared to controls. Nevertheless, once blood flow was normalized for limb muscle mass, evidence turned in favor of a preserved vascular function in SCI. Olive et al. (Olive et al. 2003) also measured the common femoral artery diameter and hyperemic response to thigh limb occlusion in individuals with SCI and a control group. Again, diameter and blood flow resulted significantly diminished in SCI compared to controls. When those variables were observed per muscle volume unit, no difference was detected between SCI and controls, indicating that vascular atrophy observed in this population was closely linked to muscle atrophy (Olive et al. 2003). De Groot et al. (Groot et al. 2006) observed the time course and the magnitude of adaptations of vascular dimension and endothelial function during the first 6 weeks right after a SCI. Their results showed a rapid onset of arterial adaptation including reduction in femoral artery size and blood flow, together with a rapid decline in lower limbs muscle mass. Still, the difference between SCI and able-bodied individuals was evident only for the absolute values, as correcting for the reduced muscle volume in SCI individuals eliminated the difference in diameter and blood flow. Authors suggested that adaptations in vascular function closely parallel skeletal muscle atrophy in this population (Groot et al. 2006). Results of our study strongly confirm previous evidence supporting a link between vascular remodeling and muscle volume. Importantly, our results go beyond previous evidence since, in this case, they are not confounded by the presence of sudden lack of physical activity or denervation. Also, we measured all variables potentially involved in, or resulting from, vascular remodeling, such as blood flow, blood velocity, shear rate, vascular conductance, and arterial stiffness, but none of them was different between the two limbs indicating that in one-leg amputee model, the only variable that could have a role in vascular remodeling seems to be muscle mass.

### Physiological consideration on the link between vascular remodeling and muscle mass

Needless to say, blood vessels are no longer considered to be simply passive conduits for blood flow. Continuous evidence emphasizes their capacity to efficiently adapt to maintain optimal function in response to continually changing hemodynamic and metabolic conditions (Folkow 1971; Gibbons and Dzau 1994). In this view, it has to be considered that physiologically, different vascular circuits are specifically designed to meet the particular demands of their tissues, both as to the range of blood supply and how this supply is distributed and put into efficient exchange within the tissue (Folkow 1971). Therefore, it is not difficult to conceive that once tissues around vessels change in volume and/or composition, a similar change in vascular structure and function may take place, based on the variation in amplitude of the metabolic demand. A non-reversable reduction of muscle mass, such as in the case of amputee individuals, brings with it a reduction of vascular mass in terms of rarefaction of the microcirculation (a loss of capillary area from the lost tissues), indirectly stimulating changes in the architecture of bigger vessels responsible for the perfusion of that area (Betz et al. 2021; Gibbons and Dzau, 1994). In fact, a significant reduction in capillary bed implies a variation of hemodynamics with a significant sudden change in shear rate. In response to this event, arteries that are genetically programmed to continuously regulate their lumen to maintain constant wall shear stress at a preferred homeostatic value, first decrease their caliber acutely via a vasoactive response (Gibbons and Dzau 1994; Humphrey 2008). When the altered flow is sustained, the change in arterial caliber is maintained by remodeling the extracellular matrix and smooth muscle of the wall at the new, smaller, diameter (Humphrey 2008).

Findings of our study suggest that muscle mass is a dominating factor playing a pivotal role in vascular remodeling. To guarantee a proper vascular structure and function, muscle mass must be maintained, avoiding negative modification in microcirculation that would serve as remodeling stimuli for arterioles and conduit arteries. From this point of view, knowing that muscle mass cooperates with vasculature, new strategies that include positive modification of muscle volume can be explored to develop efficient strategies that support vascular structure, function, and health in specific populations characterized by negative vascular modifications.

## Limits of the study

The current study has some limitiations. First, although individuals included in the study declared to be physically active (i.e.,actively involved in team sports training at least three times a week), objective measurement of amount of physical activity by means of questionnairs, steps count or othe fitness testing have not been performed. Second, how long participants have been active since amputation is another missing information which might have been helpful in supporting the results of this study.

## Conclusions

Results from this study confirm previous findings about the role of muscle mass in vascular remodeling. However, to our knowledge, this is the first study that investigates exclusively muscle mass in relation to vascular structure and function, without using a model encompassing physical inactivity and denervation. Interestingly, using a single-leg amputee human model, where the amputee leg maintains its innervation and it is still used for locomotion, the only variable related with the reduction of arterial diameter was muscle mass, and no other hemodynamic variables such as shear rate, and blood flow. These finding may help in developing further study to better understand vascular adaptation and the mechanisms involved in this phenomenon and the interaction between each other.

## Data Availability

Data are available upon request to the corresponding author.
